# Benthic reef assemblages of the Fernando de Noronha Archipelago, tropical South-west Atlantic: Effects of depth, wave exposure and cross-shelf positioning

**DOI:** 10.1371/journal.pone.0210664

**Published:** 2019-01-10

**Authors:** Zaira Matheus, Ronaldo Bastos Francini-Filho, Guilherme Henrique Pereira-Filho, Fernando C. Moraes, Rodrigo L. de Moura, Poliana S. Brasileiro, Gilberto Menezes Amado-Filho

**Affiliations:** 1 Instituto de Pesquisas Jardim Botânico do Rio de Janeiro, Rio de Janeiro, RJ, Brazil; 2 Departamento de Engenharia e Meio Ambiente, Universidade Federal da Paraíba, Rio Tinto, PB, Brazil; 3 Laboratório de Ecologia e Conservação Marinha, Instituto do Mar, Universidade Federal de São Paulo, Campus Baixada Santista, Santos, SP, Brazil; 4 Instituto de Biologia, Laboratório de Sistemas Avançados de Gestão da Produção (SAGE), Instituto Alberto Luiz Coimbra de Pós-Graduação e Pesquisa de Engenharia (COPPE), Universidade Federal do Rio de Janeiro, Rio de Janeiro, RJ, Brazil; University of California Santa Cruz, UNITED STATES

## Abstract

Oceanic islands can be relatively isolated from overfishing and pollution sources, but they are often extremely vulnerable to climate and anthropogenic stress due to their small size and unique assemblages that may rely on a limited larval supply for replenishment. Vulnerability may be especially high when these islands bear permanent human populations or are subjected to regular or intermittent fishing. Since the late 1970's, Brazil has been establishing marine protected areas (MPAs) around its four oceanic island groups, which concentrate high endemism levels and are considered peripheral outposts of the Brazilian Biogeographic Province. In 2018, the Brazilian legally marine protected area increased >10-fold, but most of the ~1,000,000 km^2^ of MPAs around Brazil's oceanic islands are still unknown and unprotected. Here, we provide the first detailed quantitative baseline of benthic reef assemblages, including shallow and mesophotic zones, of the Fernando de Noronha Archipelago (FNA). The archipelago is partially protected as a no-take MPA and recognized by the UNESCO as a World Heritage Site, but also represents the only Brazilian oceanic island with a large permanent human population (3,000 people), mass tourism (up to 90,000 people per year) and a permanent small-scale fishing community. The influence of depth, wave exposure, and distance from the island and shelf edge on the structure of benthic assemblages was assessed from benthic photoquadrats obtained in 12 sites distributed in the lee and windward shores of the archipelago. Unique assemblages and discriminating species were identified using Multivariate Regression Trees, and environmental drivers of dominant assemblages’ components were evaluated using Boosted Regression Trees. A total of 128 benthic taxa were recorded and 5 distinct assemblages were identified. Distance to the insular slope, depth and exposure were the main drivers of assemblages’ differentiation. Our results represent an important baseline for evaluating changes in benthic assemblages due to increased local and global stressors.

## Introduction

The Brazilian Biogeographic Province (BBP) is a secondary biodiversity centre in the Atlantic Ocean that extends along the eastern South American margin from the Amazon mouth (04°N) south to Santa Catarina (29°S) [1]. The BBP is closely related to the Caribbean Biogeographic Province and encompasses ~690,000 km^2^ of continental shelf (<100 m depth), as well as four peripheral outposts in oceanic archipelagos with high endemism levels: St. Peter and St. Paul’s Archipelago (SPSPA), Fernando de Noronha Archipelago (FNA), Rocas Atoll (ROA), and Trindade/Martin Vaz Archipelago (TMVA) [2]. The ecological relevance of these oceanic islands motivated the declaration of no-take marine protected areas (ntMPA) in ROA (1978, 35,186.41 km^2^) and FNA (1988, 10,929.47km^2^), as well as a multiple-use MPA (muMPA) in FNA, ROA and SPSPA (1986, 154,409.03km^2^). In 2018, muMPAs and ntMPAs were declared in SPSPA (400,000 and 40,000 km^2^, respectively) and TMVA (400,000 and 60,000 km^2^), this latter also covering the adjacent Columbia Seamount. Together, the MPAs around Brazil’s oceanic archipelagos cover 809,429.4 km^2^ under multiple-use and 116,418.5 km^2^ under no-take regimes, but most of their areas is below 100 m depth and remains to be characterized even in terms of bottom type and oceanographic forcing [3].

With the exception of SPSPA, which drops steeply into the deep sea, rhodolith beds dominate the relatively flat insular shelves and adjacent seamounts’ tops surrounding all of these island groups [4, 5]. Benthic assemblages of shallow (< 30 m depth) and mesophotic (30–90 m) reefs of ROA [6–8], SPSPA [9, 10] and TMVA [11] have already been characterized, but comprehensive quantitative information is still lacking for FNA [12], the only Brazilian oceanic island with significant human population and infrastructure (3,000 inhabitants).

Oceanic islands can be relatively isolated from overfishing and pollution, but they are often extremely vulnerable due to their small size, especially when they bear permanent human populations or are subjected to regular or intermittent fishing [13]. Mesophotic benthic assemblages may harbour unique biodiversity and ecological characteristics [14–16], and have been regarded as less susceptible to disturbances (the ‘deep reef refugia’ hypothesis [17, 18]). However, mesophotic reefs tend to be neglected in assessments and monitoring programs, and their responses to global changes and local impacts are still controversial [19, 20, 21]. Thus, comprehensive baseline assessments of the shallow and mesophotic reefs of the Brazilian oceanic islands [4, 8, 9, 11, 22] are essential for long-term monitoring aiming to assess the ecological processes underlying reef dynamics [23, 24] and MPA effectiveness.

The spatial and temporal dynamics of benthic assemblages are influenced by abiotic (e.g, depth, wave exposure and bottom inclination) and ecological drivers (e.g., competition, predation, recruitment), as well as by anthropogenic forcing (e.g. overfishing, pollution) [25, 26]. At mesophotic depths, wave exposure, temperature and light availability decrease sharply, with clear effects on benthic assemblages [9, 27, 28]. Distance offshore also influences the structure of benthic assemblages due to land-sourced nutrification and turbidity gradients, and also by constraints in larval supply. Deeper offshore sites may also be affected by nutrient-rich and colder upwelling waters enriched in zooplankton [29]. The release of top-down control due to overfishing also promotes cascading effects and acute changes in benthic assemblages [30]. Therefore, the influence abiotic and biotic drivers is hard to disentangle and often results in complex spatial and temporal patterns [31, 32]. Multivariate Regression Trees (MVRT) are a powerful tool to deal with assemblage data, as they account for non-linear relationships, continuous and categorical data, missing values, imbalance and complex interactions. They can identify discrete assemblages along environmental gradients and are easy to interpret [31, 32]. In this context, we used MVRT to identify different benthic reef assemblages and their main drivers across a wide depth gradient (5–50 m) in the FNA. In addition, univariate patterns of the most abundant species/functional groups were also described. The results obtained here constitute a powerful baseline that will help understand possible global and local impacts on reef assemblages and support management and conservation actions in this important South Atlantic Oceanic reefs.

## Material and methods

### Study area

**Fig 1:**
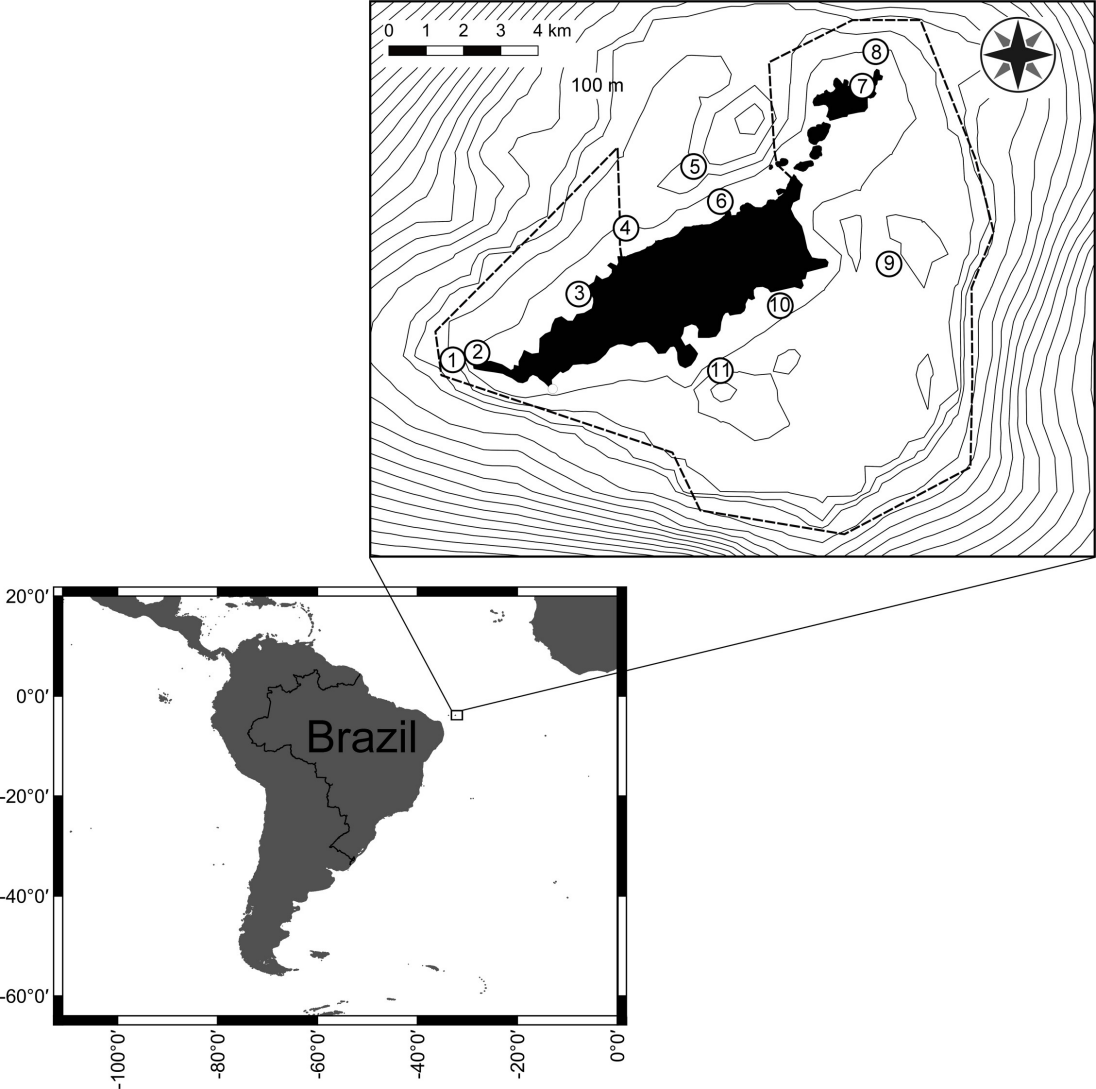
Map of the Fernando de Noronha Archipelago. Sampling sites: 1 –Cabeço da Sapata (CBS), 2 –Caverna da Sapata (CVA), 3 –Baía dos Golfinhos (BG), 4 –Laje Dois Irmãos (LDI), 5 –Cabeço dois Irmãos (CDI), 6 –Morro de Fora (MF), 7 –Buraco do Inferno (BI), 8 –Cabeço das Cordas (CC), 9 –Pedras Secas (PS), 10 –Ilha do Frade (IF) and 11 –Cabeço Submarino (CSU).

Fernando de Noronha is a volcanic archipelago located on the relatively flat top of a seamount within the Fernando de Noronha Ridge (Fig 1). The region is under the influence of the west-flowing, warm (26–27 C°), saline (36°/_oo_), and oligotrophic Equatorial Current [33]. It encompasses 20 islands and islets that total ~27 km^2^, and ~190 km^2^ of insular shelf (<80m) dominated by rhodolith beds, with soft sediments nearshore [5]. Rocky reefs occur largely as the islands' shore in depths of up to 60 m, and also as a few isolated outcrops across the shelf. The upper slope (90–70 m depth) is rocky and steep, with low sediment accumulation. Carbonate framework dominated by coralline algae occurs as incipient fringes in the shallower part of the islands (<20m) and around outcrops, especially on the windward shore [5, 34, 35]. Climate is tropical with predominance of SE/E trade winds during the summer. The leeward (NW) shore is known as “mar de dentro”, while the windward (SE) shore is known as “mar de fora” (Fig 1). There are only a few intermittent freshwater streams, but FNA encompasses the only mangrove habitat in Brazilian oceanic islands (0.01 km^2^) [12].

### Sampling

We used SCUBA with mixed-gas to sample 12 sites in the lee- and windward shores of FNA, in November 2011, covering different exposures, depths, and distances offshore and to the slope (Fig 1). Benthic assemblages were characterized using haphazardly placed photo-quadrats (n = 221) in five depth strata: 0–10, 10–20, 20–30, 30–40, 40–50 m (n = 5–15 per stratum). After the divers have reached the depth strata, photo quadrats were placed by them non-intentionally in intervals of 10 diver fin kicks. Photo quadrats were composed of a mosaic of 15 high-resolution digital images totalling 0.3 m^2^, see Magalhães et al. [9] for details. Relative cover was estimated through the identification of organisms at the lowest possible taxonomic level below 300 randomly distributed points per quadrat using the Coral Point Count with Excel Extensions Software [36]. Turf algae were defined as “<5 cm high multispecies assemblages structured by short algae, mostly with filamentous or smaller-sized corticated morphology” cf. [9, 37].

Hydrodynamism was evaluated from weight loss of gypsum balls attached to a vertical line and a buoy for 24h. A total of 60 units were deployed at the windward (n = 30) and leeward shores (n = 30) (Fig 1). Once wind is always stronger in the windward portion of the FNA, gypsum balls were deployed only in October 2011, a month that represents intermediate conditions between summer swells and winter relatively calmer seas. The units were weight before and after deployment to the nearest 0.01g [38, 39].

### Data analyses

Differences in weight loss of gypsum units between sites were tested using Analysis of Variance (ANOVA) followed by Tukey *post-hoc* tests [40]. Multivariate Regression Trees (MVRT) were used to identify unique benthic assemblages and the main environmental drivers leading to their differentiation. A MVRT is built by repeatedly splitting groups of samples aiming at producing nodes as homogeneous as possible regarding the response variable. Homogeneity is measured by finding the best split that minimizes the sums of squares about the multivariate mean within each node [32]. The MVRT approach makes no assumptions about the relationships between dependent and independent variables and an optimal tree is selected based on its predictive accuracy through cross validation. Accuracy is inferred from the cross-validation correlation, which varies from zero (poor predictor) to one (excellent predictor) [32]. Another approach is to make a PCA with different MVRT solutions in order to identify the one best explaining variability in the data. For the MVRT analysis, unidentified species within major taxa (e.g. sponges) were pooled into a single category.

Boosted Regression Trees (BRT) were used to understand drivers of abundance of the most common benthic organisms (> 2% relative cover) and the ones contributing to splitting (i.e. assemblage differentiation) on the MVRT model, with 10 taxa/functional groups retained for the final analyses (see Results). The BRT models were built following the procedures of [31]. The basic BRT approach consists on the combination of a large number of simple regression trees (in which predictions are based on recursive binary splits) using the technique of boosting in order to improve model accuracy. At each step, a new tree is fitted for the residuals from the previous model aiming at reducing the loss function. The process is stochastic, as only a subset of the original data, set at random, is used at each step. The most important attributes of BRT models are bag-fraction, learning rate and tree complexity. Bag-fraction is the proportion of data selected to fit a tree at each step (e.g. a bag-fraction of 0.5 means that 50% of the data are selected at random to fit a tree). The learning rate determines the contribution of each tree to the overall model explanation, while the tree complexity represents the number of nodes (splits) of each tree [31, 32]. To avoid model overfitting and attain highest accuracy, as indicated by lowest values of cross-validation deviance and standard error, optimal BRT models were selected by examining all possible combinations of values for bag-fraction (0.5 and 0.75), learning rate (0.001, 0.005, 0.01 and 0.05) and tree complexity (1 to 5) cf. [31].

All analyses were carried out in R (version 3.2.5; R Core Development Team 2014; available at http://www.R-project.org). Data from this work was made available for public access through the Dryad platform (http://datadryad.org/).

## Results

**Fig 2:**
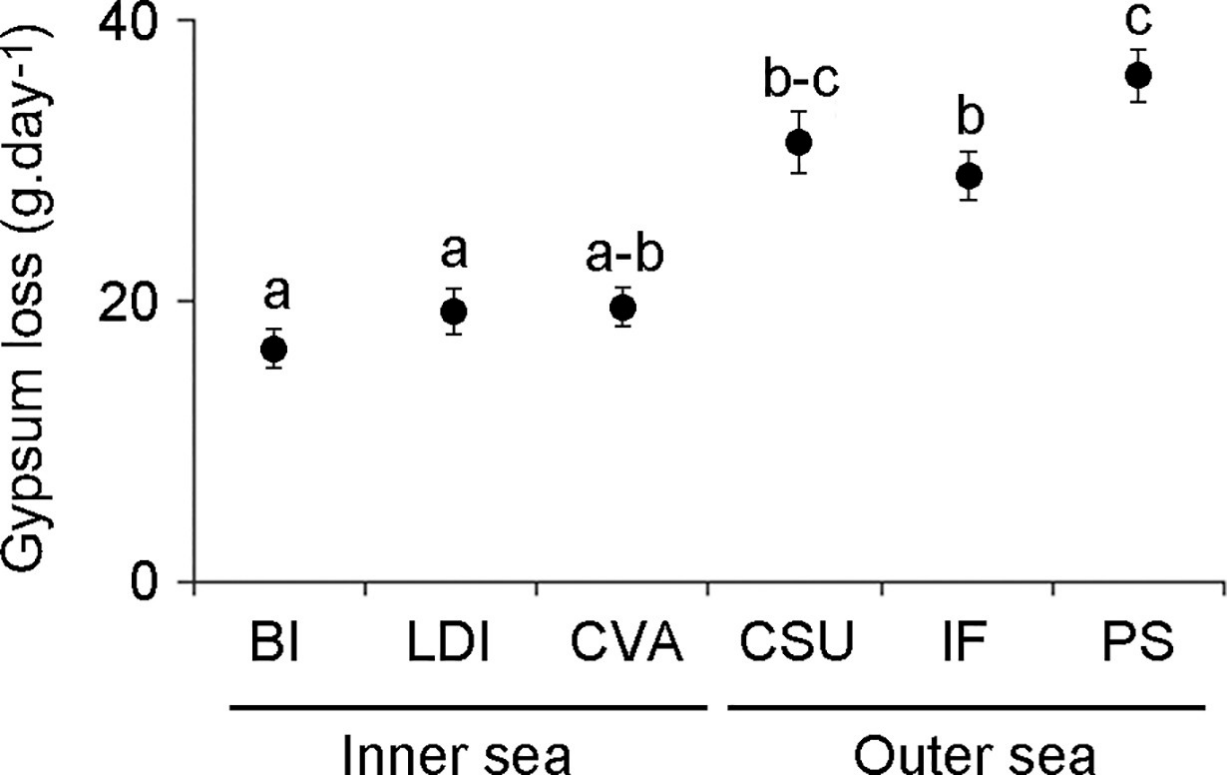
Mass loss (mean ± SE) of gypsum units deployed in the inner (sheltered / NW) and outer (exposed / SE) sides of the FNA. Letters indicate differences detected by Tukey *post hoc* test. Codes for sampling stations as in Fig 1.

As expected, higher turbulence (as inferred from gypsum ball mass loss) was recorded in the SE side of the FNA than in the NW side (ANOVA: p < 0.05) (Fig 2). A total of 128 benthic taxa belonging to eight major groups (cyanobateria, fleshy macroalgae, turf algae, crustose calcareous algae, Porifera, Hydrozoa, Zoanthidea, Anthozoa and Ascidiacea) were recorded. Porifera was the most speciose group, with 69 infrageneric taxa, followed by fleshy macroalgae (34 taxa). Seven species of scleractinian corals were also recorded (S1 Table). Most abundant benthic organisms (> 2% of total benthic cover) were as follows: the scleractinian coral *Montastrea cavernosa* (11.6% of total benthic cover), crustose calcareous algae (CCA; 9.5%), the algae *Dictyopteris jamaicensis* (8.8%) and *Canistrocarpus cervicornis* (8.7%), sediment (7.1%), *Caulerpa* ssp. (6.2%), turf algae (5.2%), the sponge *Monanchora arbuscula* (4.8%), *Jania*/*Amphiroa* algae (4.4%) and the fire-coral *Millepora alcicornis* (2.3%).

**Fig 3:**
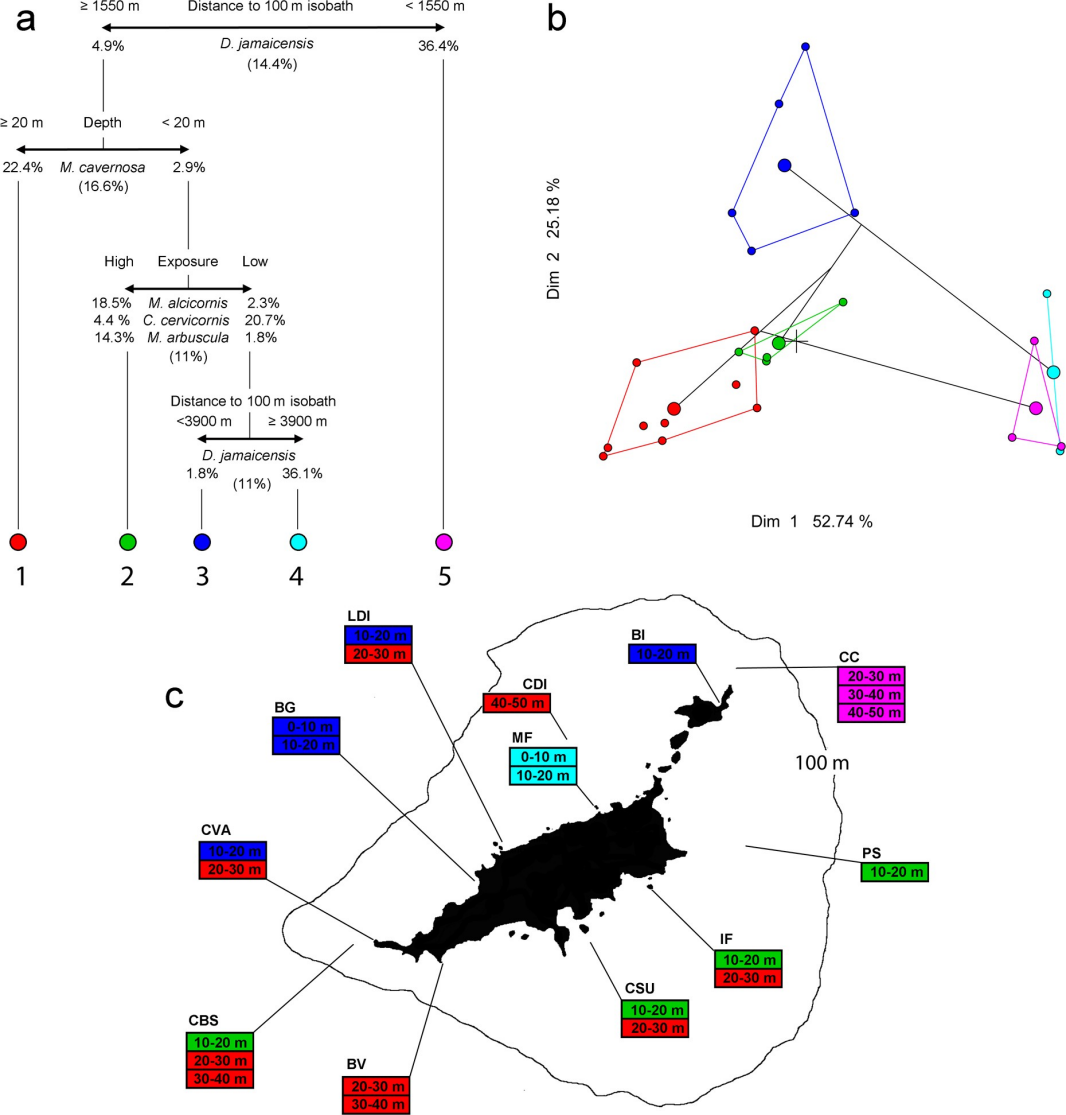
Benthic reef assemblages of the Fernando de Noronha Archipelago. (A) Multivariate Regression Tree (MVRT) for benthic assemblages of the Fernando de Noronha Archipelago, off NE Brazil. Environmental drivers for each split are given in the upper portion, while organisms responsible for the split and the relative contribution of the split to total model variation explained are given in the lower portion. (B) Principal Component Analysis (PCA) plot showing separation between the five distinct assemblages identified by the MVRT. (C) Spatial distribution of the five typical assemblages according to depth strata and sampling sites. Codes for sampling stations as in Fig 1.

**Fig 4:**
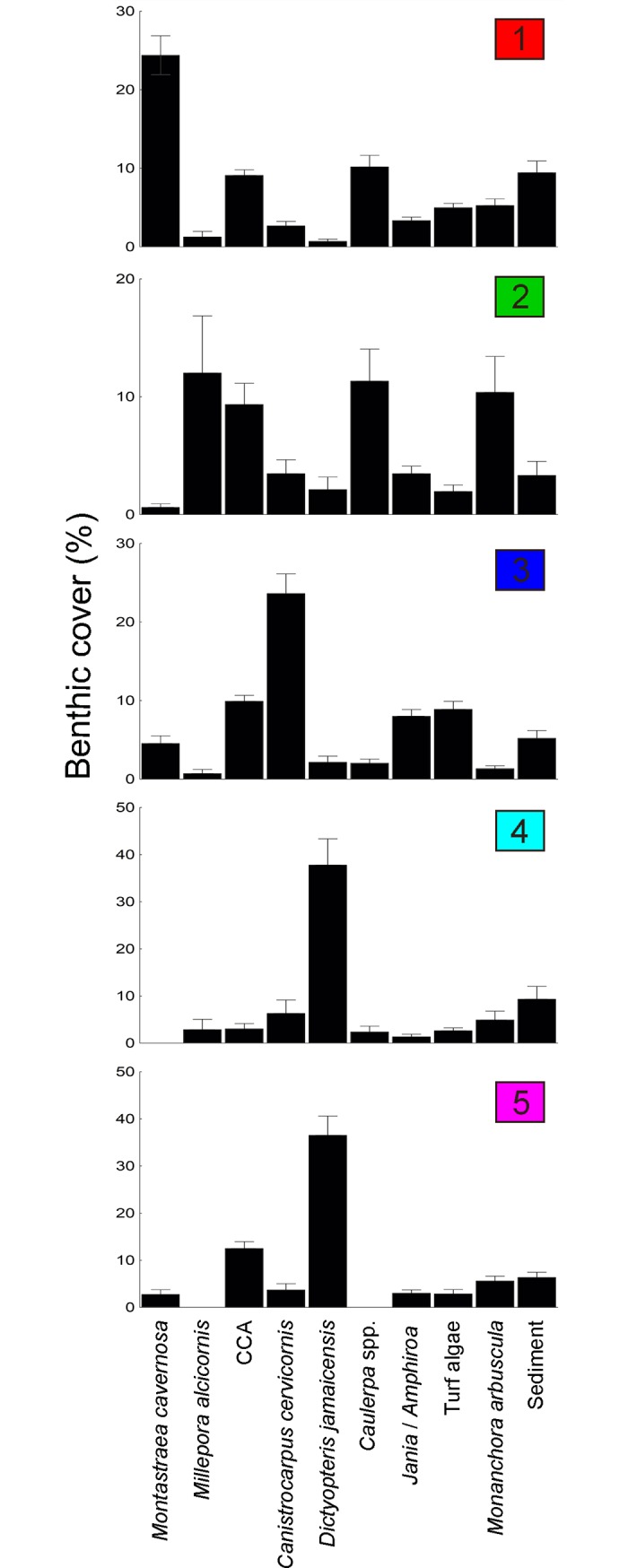
Relative abundance (mean ± SE) of species/functional groups responsible for splits (i.e. differentiation between assemblages) in the MVRT model in the five typical assemblages. All species/groups representing > 2% of total benthic cover are shown. Colour pattern follows Fig 2. Note differences in scale of y axis.

The best MVRT (cross-validated relative error = 0.47 and cross-validation correlation = 0.53) identified five distinct benthic assemblages within the FNA (Fig 3A). The first two axes of the PCA calculated considering these five assemblages explained 77.9% of total variability in the data (Fig 3B). The first split of the MVRT separated a group of four assemblages characterized by low *Dictyopteris jamaicensis* cover (<4.9%) from a single assemblage characterized by high *D*. *jamaicensis* cover (>36.4%) (Fig 4). This latter assemblage was found at all three depth strata (20–50 m) from the Cabeço das Cordas site. The main environmental driver responsible for this first split was the distance from the insular shelf break (i.e. 100 m isobath). Cabeço das Cordas was the closest site to the insular shelf break (< 1550 m), while all other assemblages/sites were located > 1550 m from deep reefs. The second split distinguished a relatively deep assemblage (20–50 m) characterized by high *M*. *cavernosa* cover (mean 22.4%) (located in both, the leeward and windward) from three shallow assemblages in the inner and outer sea, all of them with low *M*. *cavernosa* cover (2.9%) (Fig 4). This latter group was further subdivided according to exposure, with one assemblage composed by one homogeneous assemblage of shallow high exposed reefs (10–20 m) in the outer sea (i.e. surge zone) and two other assemblages in shallow (0–20 m) low exposed reefs of the inner sea. Reefs in the surge zone of the outer sea were dominated by *M*. *alcicornis* and *M*. *arbuscular*. The two shallow assemblages in the inner sea were characterized by low (1.8%) and high (36.1%) *D*. *jamaicensis* cover, the former in sites closer to deep reefs and the latter in the a single site closest to the island (Morro de Fora) (Fig 3C).

The main environmental drivers, spatial and bathymetric distribution of assemblages and the relative cover of dominant and typical taxa/functional groups of each of the five benthic assemblages within the FNA are summarized in Figs 3 and 4.

**Fig 5:**
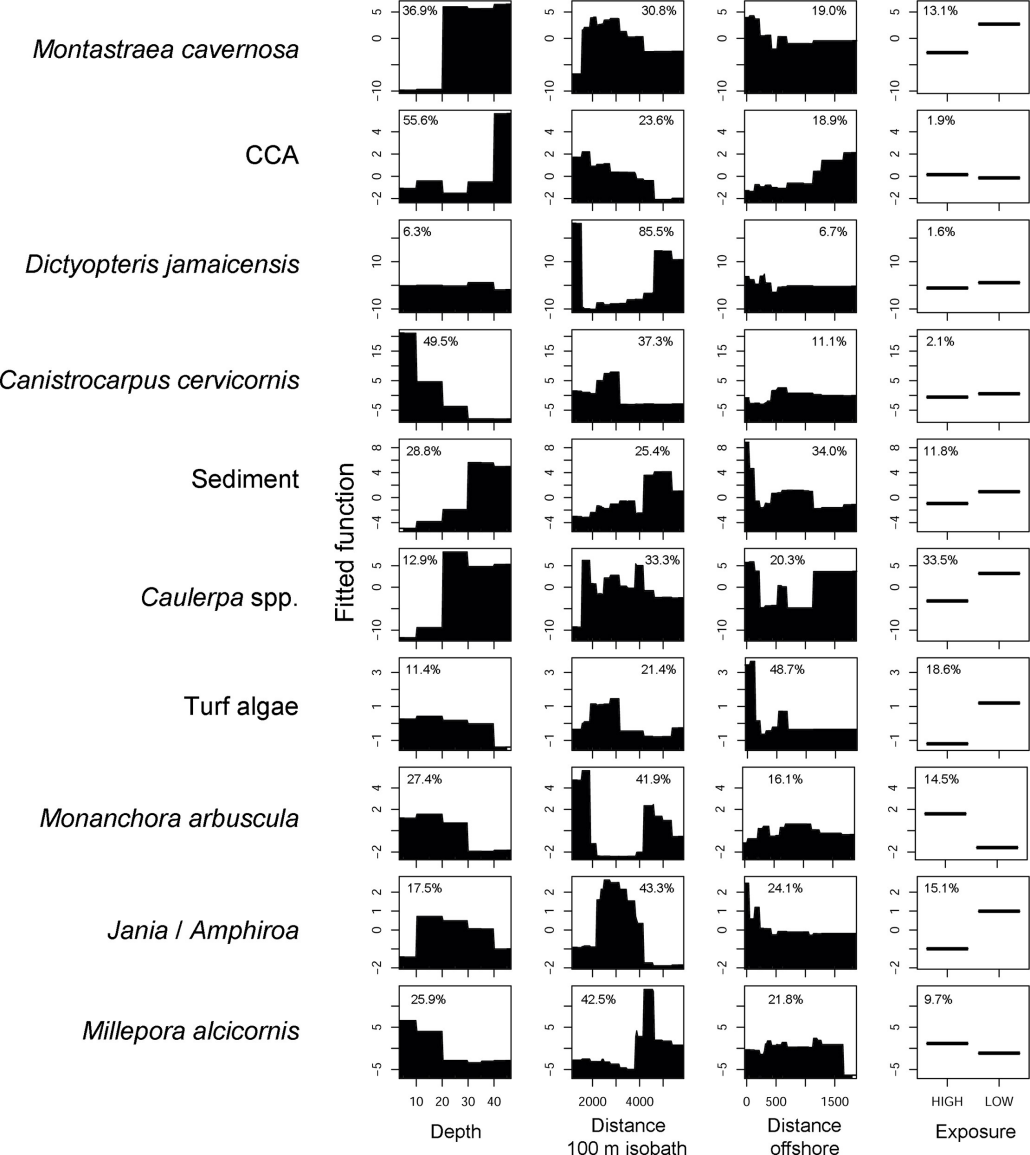
Partial dependence plots obtained with Boosted Regression Trees using four predictors (depth, distance from 100 m isobath, distance offshore and exposure). Relative contributions (%) of explanatory variables are given. Taxa/functional groups are shown in decreasing order of abundance. Only the most abundant taxa/functional groups (> 2% of total benthic cover) and the ones responsible for differentiating benthic assemblages (see Fig 2A) are given. Y axes are centered to have zero mean over the data distribution.

The BRT models supported the patterns observed in the MVRT, but added more detail to the main environmental drivers shaping the abundance and spatial distribution of the most abundant components of benthic assemblages (Fig 5 and Table 1). Depth was the most important predictor for *M*. *cavernosa*, CCA and *C*. *cervicornis*. *M*. *cavernosa* dominates in depths > 20 m and CCA is most abundant in depths greater than 40. On the other hand *C*. *cervicornis* dominates reefs shallower than 20 m. Distance to deep reefs was the main environmental driver for variation in abundance of *D*. *jamaicensis*, *M*. *arbuscula*, *Jania*/*Amphiroa* and *M*. *alcicornis*. Abundance of the former two species was lower at intermediate distances, and the opposite pattern was recorded for *Jania* / *Amphiroa*. *Millepora alcicornis* was more abundant in reefs > 4000 m from deep reefs.

**Table 1:** Optimal settings and predictive performance of boosted regression tree (BRT) analyses used for modeling spatial patterns in abundance (relative cover) of dominant benthic organisms/functional groups (> 2% of total benthic cover; decreasing order of abundance) in the Fernando de Noronha Archipelago, off NE Brazil. Optimal settings: bf–bag fraction, lr–learning rate, tc–tree complexity.

BRT model	Optimal settings			Number of trees	Cross Validation Deviance (± SE)	Cross Validation Correlation (± SE)
	*bf*	*lr*	*tc*			
*Montastraea cavernosa*	0.5	0.001	4	4550	201.4 (23.0)	0.66 (0.02)
Crustose Calcareous Algae	0.75	0.001	3	6200	41.4 (4.7)	0.46 (0.03)
*Dictyopteris jamaicensis*	0.5	0.005	5	2050	123.8 (21.6)	0.80 (0.03)
*Canistrocarpus cervicornis*	0.75	0.001	4	4950	96.8 (23.0)	0.73 (0.05)
Sediment	0.75	0.005	4	5500	76.1 (12.9)	0.59 (0.10)
*Caulerpa* spp.	0.75	0.001	5	3000	91.9 (10.8)	0.52 (0.05)
Turf algae	0.75	0.001	2	4350	29.3 (3.8)	0.51 (0.06)
*Monanchora arbuscula*	0.75	0.001	4	4650	47.2 (8.0)	0.53 (0.07)
*Jania* / *Amphiroa*	0.75	0.001	4	3450	18.8 (2.8)	0.53 (0.08)
*Millepora alcicornis*	0.75	0.005	4	6500	65.0 (29.2)	0.54 (0.10)

Distance offshore was the most important predictor for turf algae and sediment abundance, with both dominating in reefs < 200 m from the island. Exposure was the main environmental driver for *Caulerpa* spp. cover, which showed higher abundance on low exposed areas, with minor effects on other organisms (Fig 5).

## Discussion

This is the first comprehensive quantitative assessment of benthic rocky reef assemblages of the FNA since the seminal work by [12]. The main methodological difference between [12] and this one is that the former focused on shallow reefs (< 30 m depth), while we focused on a broader depth gradient (0–50 m). Eston et al [12] describe benthic assemblages strikingly dominated by fleshy macroalgae (50–100% cover), particularly *Dictyota* spp., *Dictyopteris* spp. and *Sargassum* spp., in their three shallow stations (Buraco do Inferno, Carreiro da Pedra and Desembarque sites). Within these three stations, samples were obtained in platforms < 10 m depth and maximum depths of < 15 m (see Figs 3–5 in [12]). The only site in which [12] found a relatively high cover of Porifera and scleractinian corals (particularly *M*. *cavernosa*) was the deepest site sampled by them (Caverna da Sapata, 30 m maximum depth). The Caverna da Sapata site was also sampled here, with the identification of two distinct assemblages: one between 10–20 m dominated by the fleshy macroalga *C*. *cervicornis* (formerly *Dictyota cervicornis*) and another between 20–30 m dominated by *M*. *cavernosa*. Thus, the general pattern of fleshy macroalgae dominance (particularly *Dictyopteris jamaicensis* and *C*. *cervicornis*) in shallow stations (< 20 m) and dominance of *M*. *cavernosa* in deeper stations (20–50 m) recorded here, broadly agrees with patterns described by [12]. However, only a systematic monitoring program may help to elucidate possible temporal shifts in the structure of benthic reef assemblages of the FNA and the present work represents an important baseline for future comparisons.

Turf and fleshy macroalgae are dominant components of shallow benthic rock reef assemblages of the four Brazilian oceanic islands [4, 6, 7, 9, 11, 12]. Algal predominance may be driven by overfishing of herbivores and eutrophication, being thus considered as a symptom of reef degradation [30, 41, 42]. However, there are indications that natural factors, such as high light intensity and low herbivory levels, may lead to algal predominance in some habitats [43, 44]. For the FNA there is no indication of overfishing, as most of the Archipelago is fully protected and herbivorous fishes are abundant [35]. Future studies may clarify if natural factors and/or pollution (sewage) are driving high macroalgae abundance. In addition, using Pacific and Caribbean reefs as global baselines may have several limitations, as they are, most likely, not representative of the reefs of other regions [9, 44]. In fact, algal beds have already been recognized as important nursery habitats for some reef fishes in NE Brazil [45]. High algal abundance in inner-shelf reefs of the FNA could be driven by low herbivory intensity and indeed biomass of large herbivorous fishes is relatively low on shallow reefs of the FNA [35]. Unfortunately, cross-shelf patterns in herbivory intensity *per se* were never measured in continental and insular Brazilian reef ecosystems. Experimental studies may help to elucidate the relative contribution of top down (herbivory) and bottom-up (light and nutrient availability) processes leading to high turf and macroalgae abundance in Brazilian oceanic islands.

Five distinct benthic assemblages were identified in the MVRT. Distance to the break of the insular shelf (100 m isobath), depth and exposure were the main environmental drivers leading to assemblages’ differentiation. A “typical” cross-shelf pattern was recorded, with high turf and macroalgae abundance in shallow inner-shelf reefs, and high abundance of reef building organisms (particularly crustose calcareous algae and the scleractinian coral *Montastraea cavernosa*) in deeper outer-shelf reefs [29]. The BRT models corroborated the assemblage-level cross-shelf pattern recorded in the MVRT, as depth and distance offshore were among the most important predictors for most species/functional groups. Sediment was particularly abundant in sites close to the island (< 200 m), suggesting sediment may facilitate algae abundance and negatively influence CCA and coral abundance on inner-shelf reefs of the FNA. The site closest to deep reefs (Cabeço das Cordas) harboured a unique benthic assemblage across a broad depth gradient (20–50 m) characterized by the highest CCA cover within the FNA (see Fig 4). Lower water temperatures due to upwelling, steeper slopes, less sediments, and distance from the island are among the alternative non-exclusive hypotheses that may explain the influence of proximity to deep reefs on benthic assemblages. Proximity to the continental shelf break has been previously demonstrated to influence the dynamics of reef fish assemblages of the Abrolhos Bank, Brazil [46], highlighting the importance of including this variable in future studies on reef communities.

Shallow stations (10–20 m depth) off the exposed side of the FNA harbour a typical assemblage characterized by relatively high abundance of the *Caulerpa* spp., the fire-coral *Millepora alcicornis* and the sponge *Monanchora arbuscula*. In addition, one algal species (*Turbinaria turbinata*) occurred exclusively in the 10–20 m depth stratum of Pedras Secas, the second most abundant organism in this site (18.3% relative cover). *Turbinaria turbinata* is typical of turbulent waters [47] and indeed Pedras Secas was the site with highest turbulence within the FNA (see Fig 2). *Caulerpa* spp. (particularly *C*. *racemosa* var. *peltata*) were major components of shallow (5–10 m depth) benthic assemblages of the St. Peter and St. Paul’s Archipelago, which are also subjected to strong turbulence [9]. The latter results corroborate the hypothesis that the *Caulerpa* genus can survive under high disturbance regimes [48]. High ecological versality and ability to thrive in shallow high hydrodynamical environments are also known for *M*. *alcicornis* [49] and *M*. *arbuscula* [50].

Despite logistical challenges, the assessment of mesopohotic reefs of geographically isolated oceanic sites in Brazil increased sharply in the last decade, e.g., [5, 6, 7, 8, 9, 11, 22, 50]. Although the widely accepted shallower limit of the mesophotic zone is 30 m [27, 28], results obtained here indicate a single benthic assemblage in the upper mesophotic zone of the FNA between 20–50 m (note the lower limit of this assemblage may be deeper, as no samples were obtained > 50 m depth). Upper mesophotic reefs of the FNA were dominated by the scleractinian coral *M*. *cavernosa*. This same coral species is the most abundant scleractinian of the largest coral reef complex in the South Atlantic (Abrolhos, central Brazilian coast), corroborating the idea that it is the current major reef-building species in the SW Atlantic [51] and highlighting the importance of further studies focusing on its conservation status. In the Caribbean *Montastraea* reefs support high biodiversity and a large suite of ecosystem processes and services [52]. *Montastraea cavernosa* is recognized as a sediment resistant species [53] and is associated with both shallow high turbidity coastal reefs [51] and low light sediment-free mesophotic reefs [11, 28]. Another important aspect of mesophotic reefs of the FNA was the relatively high richness of Porifera species. Most sponge species recorded here (67 taxa), including possible new occurrences and species (see S1 Table), occurred between 40–50 m. This is noteworthy, as the FNA was already previously recognized as hosting the highest richness of Porifera among Brazilian oceanic islands [54].

This study adds to the knowledge about Brazilian oceanic islands providing important baseline quantitative data for shallow and mesophotic benthic reef assemblages of the FNA. Although protected from fishing, the growing population of the FNA (from about 2000 to 6000 people in the last 20 years) may lead to potential indirect local impacts (e.g. pollution). In addition, the global trend of increased sea surface temperature and acidity [55–56] highlights the importance of implementing a long-term monitoring program focusing on reef assemblages of the FNA.

## Supporting information

S1 Table Presence/absence data for benthic taxa found at diferente sites and depth strata across the Fernando de Noronha Archipelago, NE Brazil. The “X” denotes presence of the biological groups.
(XLSX)
